# O02 Cancer, Covid and control of RA – a toxic combination?

**DOI:** 10.1093/rap/rkab067.001

**Published:** 2021-10-19

**Authors:** Sharmin Nizam

**Affiliations:** Mid Yorkshire NHS Trust, Wakefield, United Kingdom

## Abstract

**Case report - Introduction:**

This case highlights the dilemma of keeping rheumatoid arthritis disease under control in active cancer cases and establishing a consistent multidisciplinary dialogue during a pandemic and staffing crises.

During chemotherapy and active cancer treatment, disease-modifying therapies (conventional and biologic) are often stopped. In some cases, the potential benefits versus risks of restarting usual therapies have to be balanced against risks of suppressing disease activity with high-dose steroids. Risks of infection (common and atypical) need to be considered.

**Case report - Case description:**

A is a 67-year-old female non-smoker diagnosed with seropositive rheumatoid arthritis (RF, anti –CCP positive) in 2008. Other conditions include type 2 diabetes, atrial fibrillation (on warfarin), hypothyroidism and obstructive sleep apnoea. Due to active disease, despite triple therapy (methotrexate, sulphasalazine and hydroxychloroquine), anti-TNF therapy (etanercept) commenced in 2009 with primary non-response. However, she responded well to B-cell therapy (rituximab) in conjunction with oral methotrexate (25mg weekly) receiving annual infusions from 2010 to 2016.

In 2017, an elective sleeve gastrectomy procedure for high BMI was abandoned after peritoneal deposits of concern were noted.  Histology and CT imaging were consistent with a primary peritoneal malignancy (Stage 3c low-grade serous adenocarcinoma). Treatment involved debulking surgery (total abdominal hysterectomy, bilateral salpinoophorectomy, omentectomy) and tamoxifen.

Treatment for rheumatoid arthritis stalled during this period but as frequent steroids were required for active joint inflammation, in agreement with the oncologists, she had a rituximab cycle in 2018.

Unfortunately, in 2019 she had signs of cancer progression (elevated tumour markers, CT imaging) and has subsequently started carboplatin chemotherapy. She has been unable to continue methotrexate or rituximab pending completion of the chemotherapy cycles (ongoing). However, her arthritis is now uncontrolled without increased steroids. Due to recurrent flares, her maintenance dose has been increased from 5mg to 7.5—10mg prednisolone daily until we can establish if it is safe and appropriate to recommence her usual arthritis regime.

Even without disease-modifying therapy like methotrexate and rituximab, risk of infection (including atypical ones) is still significant with the combination of chemotherapy and steroids. Risk of progressive joint damage and adverse quality of life with active arthritis also needs to be considered. Staffing crises, exacerbated by COVID pandemic issues, have added to complexity of decision making and coordination of regular multidisciplinary discussions regarding treatment.

**Case report - Discussion:**

Cancer is a known association in rheumatoid arthritis patients with a twofold higher risk of lymphoma compared to the general population. Whether condition or treatment affects risk remains unclear as immune dysregulation is relevant in both autoimmunity and cancer. Paraneoplastic, recent onset arthritis, chemotherapy- or immunotherapy-induced arthralgia/arthritis are also well documented. This case had a seropositive rheumatoid arthritis phenotype quite a few years prior to cancer diagnosis. Primary peritoneal cancer is uncommon, often presenting as in this case as an incidental finding. It is usually treated like ovarian cancer

Whilst methotrexate has been implicated in lung cancer, melanoma and non-Hodgkin lymphoma, overall safety data suggest any risk is quite low (e.g., EBV-associated lymphoproliferative disorders usually resolve with drug discontinuation). It is also a known chemotherapeutic agent. Anti-TNF treatment algorithms generally exclude patients with recent cancer. Rituximab, originally developed as a cancer drug, is not thought to affect risk of cancer development or progression.

Treatment with disease-modifying therapy (conventional and biologics) is often withheld in patients with active malignancy undergoing chemotherapy due to a theoretical risk of potentiated immunosuppression and toxicity, particularly cytopaenias. However, maintaining arthritis control with glucocorticoids also has short- and long-term risks. Combining chemotherapy agents like carboplatin with methotrexate has been used for urothelial carcinoma and can be well tolerated with close monitoring of haematological parameters.

Thus, it could be argued this patient is at risk of infections whichever treatment approach is taken and regaining control of arthritis with recommencement of methotrexate and rituximab is much better for her quality of life.

Regular multidisciplinary discussions are important to outline risks versus benefits of combined treatment. This may be difficult in practice during staffing crises. Covid risk in patients receiving rituximab and/or chemotherapy, timing and response to COVID vaccination are also important considerations.

**Case report - Key learning points:**

- Primary peritoneal cancer is uncommon and can present as an incidental finding
- Whilst treatment for progressive cancer is important, withholding rheumatoid arthritis treatment can have a significant adverse impact on quality of life
- Morbidity and mortality risks of stopping treatment versus combined treatment (cancer therapy and disease-modifying therapy) ideally needs to be fully discussed and agreed with the patient and all care providers – lack of “named” providers, restructuring, redeployment, multi-specialty care and a global pandemic can make coordination of this difficult

